# Health care payments in the asia pacific: validation of five survey measures of economic burden

**DOI:** 10.1186/1475-9276-12-49

**Published:** 2013-07-03

**Authors:** Sheila R Reddy, Dennis Ross-Degnan, Alan M Zaslavsky, Stephen B Soumerai, Anita K Wagner

**Affiliations:** 1 Ph.D. Program in Health Policy, Harvard University, Cambridge, MA, USA; 2 Department of Population Medicine, Harvard Medical School and Harvard Pilgrim Healthcare Institute, Boston, MA, USA; 3 Department of Health Care Policy, Harvard Medical School, Boston, MA, USA

**Keywords:** Out-of-pocket payment, Economic burden, Valid measurement, World Health Survey

## Abstract

**Introduction:**

Many low and middle-income countries rely on out-of-pocket payments to help finance health care. These payments can pose financial hardships for households; valid measurement of this type of economic burden is therefore critical. This study examines the validity of five survey measures of economic burden caused by health care payments.

**Methods:**

We analyzed 2002/03 World Health Survey household-level data from four Asia Pacific countries to assess the construct validity of five measures of economic burden due to health care payments: *any health expenditure*, *health expenditure amount*, *catastrophic health expenditure*, *indebtedness*, and *impoverishment*. We used generalized linear models to assess the correlations between these measures and other constructs with which they have expected associations, such as health care need, wealth, and risk protection.

**Results:**

Measures of impoverishment and indebtedness most often correlated with health care need, wealth, and risk protection as expected. Having any health expenditure, a large health expenditure, or even a catastrophic health expenditure did not consistently predict degree of economic burden.

**Conclusions:**

Studies that examine economic burden attributable to health care payments should include measures of impoverishment and indebtedness.

## Background

The hazardous illness-poverty trap has engendered concern over the economic and health consequences of out-of-pocket health spending [1-4]. In low and middle-income countries health expenditures are mostly out-of-pocket [5] and constitute significant portions of household spending. Public sector care providers often charge user fees to generate revenue [6]; perceived quality problems in public facilities prompt households to seek care in the more expensive private sector [1,7-9]; and insurance coverage is low. The need for large out-of-pocket health care payments threatens health care affordability and access, and impacts household economic stability and well-being. Effective measurement and monitoring of this type of economic burden is therefore necessary to inform the proper design of health financing systems.

Health care affordability studies use a variety of measures of economic burden that take into account the size of health care expense, household resources, as well as the strategy used to pay for care [1,10-12]. Studies have examined the sensitivity [10,12-14] and reliability [15] of available measures; however, to our knowledge, their construct validity has not been investigated. Construct validity, or the extent to which an indicator measures what it purports to measure [16,17], should be examined so it can be known whether such measures accurately capture the economic burden of paying out-of-pocket for health care and the degree to which households are affected by these payments. With proper validation, standard measures of health-related economic burden can be identified and used to inform policies intended to mitigate financial burden among the most vulnerable households.

To address this gap we examined the construct validity of five survey measures of different aspects of economic burden caused by out-of-pocket health care payments: 1) *any health expenditure* in which households with a health payment are compared to those without; 2) the actual *health expenditure amount* for each household; 3) *catastrophic health expenditure*, a threshold health expense above which a household’s basic standard of living is potentially endangered [10,11]; 4) *indebtedness*, whereby a household uses potentially harmful, debt inducing coping strategies to pay for care; and 5) *impoverishment*, in which a health payment pushes a previously non-poor household into poverty. Based on our findings, we discuss the utility of each measure in survey research.

Building on previous work [8], we divided the five measures into two categories: the first three measures indicate the absolute or relative *direct costs* of health care, which could affect the financial stability and basic consumption of a household [8,18]; the last two measures indicate *direct negative consequences* of health care payments, in which a household’s financial stability and livelihood have been harmed. We hypothesized that the measures of economic burden would correlate with indicators of health care need, wealth, and risk protection in the directions shown in Table 1. First, health care need is expected to be positively associated with all measures of economic burden. That is, households with an indication of need for health services (e.g., a chronically ill member) should experience greater health expenditures and incidence of catastrophic spending, indebtedness, and impoverishment than households without need. Second, wealth should correlate positively with the presence and amount of absolute health expenditures, negatively with the risk of catastrophic expenditures, and negatively with any direct negative consequences of health care payments. For example, because they have more resources, wealthier households should be able to afford higher expenditures and suffer a lower incidence of catastrophic spending, indebtedness, and impoverishment from health care payments than poorer households. Third, risk protection is expected to be negatively associated with all measures of economic burden. For example, households with health insurance should experience lower expenditures and lower incidence of catastrophic spending, indebtedness, and impoverishment than uninsured households.

**Table 1:** **Hypothesized associations between measures of economic burden and health care need**, **wealth and risk protection constructs**^**a**^

	**Measures of economic burden from health care payments**				
	**Direct**			**Direct**	
	**Cost**			**Negative consequence**	
**Construct**	**Any health expenditure**	**Health expenditure amount**	**Catastrophic health expenditure**	**Indebtedness**	**Impoverishment**
Health care need	+	+	+	+	+
Wealth	+	+	−	−	−
Risk protection	−	−	−	−	−

^a^Plus or minus signs reflect hypothesized positive or negative correlations.

These relationships are complex and may be subject to interaction effects. For instance, wealth may modify the relationship between health care need and expenditures such that poor households with illness may have, on average, relatively lower expenditures if cost-related barriers prevent them from seeking care [19]. In addition, health system factors, such as public health facility access, user fees, or insurance design, make some relationships hard to predict. For example, insured households with partial coverage of expenditures could experience higher direct costs and more frequent negative consequences than uninsured households that avoid seeking care, or that have access to free care in public facilities or through payment exemptions [19].

## Methods

Construct validity can be assessed by determining whether a measure relates to indicators of other known constructs in ways that are consistent with plausible hypotheses [16,17,20]. While a single association cannot confirm construct validity, evidence can be gathered through multiple tests of association [16,17,21]. We examined the construct validity of the five survey measures of economic burden by testing the extent to which each measure correlated with indicators of *health care need*, *wealth*, and *risk protection* in predictable ways, based on theory or research findings [2,11,22-25].

### Data source

We used cross-sectional, household-level data from the 2002/03 World Health Survey (WHS), which was developed and implemented by the World Health Organization (WHO) in 70 countries. The survey used a multi-stage cluster sampling design [26]. The same standardized questionnaire forms, data collection methods, and sampling design were used in the study countries.

### Study countries and samples

The study populations consisted of nationally representative samples of households in China, Malaysia, the Philippines, and Vietnam. Country selection was based on the following criteria: shared geographic region, adequate sample size, high response rate on household expenditure items, and variable penetration of health insurance coverage. At the time of the survey each country had some form of health insurance for parts of the population, but public health care facilities generally required user fees [6]. In China, the Philippines, and Vietnam, exemption of the poor from out-of-pocket user fees may have been inadequate [6,22,27,28]. Malaysia, according to data from an earlier period, may have had more equitable financing policies that limited user fees in public sector care and exempted the poor from payments [3,29].

Households were sampled from a nationally representative sampling frame [26,30]. With the exception of China, post-stratification weighting was performed to improve the representativeness of the sample. The interview response rate among the samples (81.4–99.7%) indicated broad coverage of the sampling frame in each country [30,31]. We excluded from the study a small percentage of households (<1%) that did not have complete annual health expenditure or survey design information. The overall study samples comprised 3,993, 6,095, 10,074, and 4,169 households from China, Malaysia, the Philippines, and Vietnam, respectively.

### Outcome measures

We defined five measures of economic burden: *any health expenditure*, *health expenditure amount*, *catastrophic health expenditure*, *indebtedness*, and *impoverishment*. We constructed the expenditure-based measures of economic burden using a variable that estimated annual health expenditure. Surveyed households reported several types of expenditures in the last four weeks: food, housing, education, health care (single-item), voluntary insurance premiums, and other goods. The health care item directed respondents to exclude expenses that would be reimbursed by insurance. In addition, households reported 4-week expenditures for eight individual health services: hospital care; outpatient care; traditional medicine; dental care; medicines; visual, hearing or prosthetic aids; diagnostic and laboratory tests; and other health care products. An additional item collected 11-month hospital expenditure. We calculated a household’s annual health expenditure by combining the annualized amounts for the eight health service items (to annualize inpatient expenditures we added the 4-week and 11-month expenses). Missing expenditure items were assumed to be zero expenditures unless all eight expenditure items were missing, in which case we instead used the annualized single health expenditure item. All household expenditure variables were converted to 2002/03 international dollars to account for the differential purchasing power of local currencies in the study countries [32].

Households had any health expense if their estimated annual health expenditure was greater than zero. The health expenditure amount was the annual health expenditure. Based on previous work, we classified households as facing potentially catastrophic health expenditures if annual health expenditure was at least 40% of annual nonsubsistence (discretionary) expenditure [2]. Households that reported borrowing money or selling assets to pay for health care in the past 12 months were classified as facing indebtedness. We defined households as facing impoverishment if they fell into poverty, that is, they were non-poor and after subtracting annual health expenditure from annual total expenditure, remaining household consumption was less than the sample-derived subsistence expenditure (i.e., the poverty line) [33]. Households classified as poor before paying for health care were, by definition, therefore excluded from the impoverishment analysis.

### Predictors and covariates

The main predictors of interest were indicators of household-level *health care need*, *wealth*, and *risk protection*. We defined households as having a health care need if a household member had a long-term illness, disability, frailty due to age, or a hospitalization in the last year. The variables hospitalization and hospital expense, although closely related, are distinct from one another; some households that experienced a hospitalization event reported they did not pay out of pocket for this care. Households were also identified as having a health care need if the survey respondent reported that he or she ever received a diagnosis of or experienced symptoms associated with a particular chronic illness (i.e., arthritis, asthma, angina, diabetes, depression, or schizophrenia) [30,34]. Household wealth was measured by a validated permanent income index that assigned households to a particular wealth quintile according to their possession of various household assets, such as furniture, appliances, or electronics [35]. This asset-based wealth variable avoids the endogeneity of expenditure-based measures of income (i.e., total consumption) when they are used to predict expenditure-based measures of burden [36]. We collapsed the wealth quintiles into two groups with the three lowest quintiles (fewer assets) as our reference group. Households were classified as having risk protection if health insurance coverage was reported for at least one member. We compared households with all members covered by insurance and households with no coverage to be certain of the coverage status among members who used health care.

To control for potential confounding, we created variables representing education, urbanicity, and household composition. We measured household education by the highest education level attained by any household member, coded as less than high school versus high school or greater. Urbanicity was determined by whether a household was located in an urban or rural setting. Household composition was represented by three variables: household size, having a member 60 years or older, and having at least one married member. Other household compositional features, such as number of children under five or females of childbearing age, were not significantly related to the outcome measures and were excluded from analyses.

### Statistical analyses

We calculated descriptive statistics for household demographic and health characteristics. We also computed proportions for each dichotomous measure of economic burden, as well as the median health expenditure amount given its skewed distribution.

We assessed construct validity using logit models to evaluate the relationships between each dichotomous outcome measure and indicators of health care need, wealth, and risk protection, summarized by logged odds ratios (log ORs). For the health expenditure amount measure we fitted a generalized linear model specified with the log link; this model produced logged ratios of health expenditures (log ERs), comparing expenditures for the binary levels of each covariate. Using the Park Test, we determined the appropriate variance function to be the variance proportional to the mean squared [37,38]. Altogether we fitted models for the five outcomes in each of the four study countries, yielding a total of 20 models, each of which included the three main predictors.

All models were adjusted for education, urbanicity, and household composition. We explored the sensitivity of the catastrophic health expenditure model to alternative definitions using a threshold of 30% or 50% of nonsubsistence spending. As a secondary analysis we stratified each adjusted model by the wealth indicator to examine the potential modifying effect of wealth on the associations between each outcome and health care need and risk protection.

We present point estimates with 95% confidence intervals for the main analyses; however, consistent with the validation literature we focus our attention primarily on the direction of these results [20,21]. We evaluated the construct validity of each measure by summarizing and comparing the number of associations across countries and constructs that were consistent with our hypotheses. Correlations were considered positive if the logged ratios were at least 0.1 and negative if the estimates were −0.11 or smaller, corresponding to ERs/ORs of 1.1 and 0.9, respectively; this decision rule allowed us to exclude marginal results. All analyses took into account the household and post-stratification weights and the clustered survey sampling design to allow for population-based inferences in each country. We conducted all analyses using Stata version 10.1 (StataCorp LP, College Station, Texas).

## Results

### Sample characteristics

Basic household characteristics for each country are exhibited in Table 2. At the time of surveys, household size was generally smaller in China and Malaysia than in the Philippines and Vietnam. Education levels were highest in Malaysia, as over 66% of households had a member who had completed at least high school. Chronic illness (16-41%) and hospitalizations (7-18%) were common in all countries. Approximately 40-75% of households across countries reported having no health insurance coverage. Nearly one-fifth of households in China and Malaysia and over one-third of households in the Philippines and Vietnam were classified as poor. In each country, total out-of-pocket spending grew with increasing wealth quintiles.

**Table 2:** **Household characteristics by country**^**a**^

	**Country**			
	**China**	**Malaysia**	**Philippines**	**Vietnam**
**Household characteristic**	**(n=3993)**	**(n=6095)**	**(n=10,074)**	**(n=4169)**
Region, (%)				
Urban	33.0	63.8	58.9	23.7
Rural	67.0	36.2	41.1	76.3
Sex distribution, (%)				
Females in household	50.3	50.6	50.1	50.8
Age distribution, (%)				
Households with children 0–5 years	16.7	35.1	45.4	28.0
Households with children 6–15 years	43.1	48.4	61.7	62.0
Households with persons 16–59 years	88.3	95.8	96.4	97.4
Households with persons 60+ years	37.3	23.2	22.4	26.4
Size, (%)				
Households with 1–2 members	22.0	24.6	9.9	7.8
Households with 3–5 members	69.8	49.1	49.7	68.4
Households with 6–10 members	8.2	25.4	38.4	23.4
Households with 11+ members	0.0	0.9	2.0	0.4
Marital status, (%)				
Households with at least one married member	91.4	81.2	82.3	93.6
Highest education by member, (%)				
No schooling or less than primary	6.6	6.7	6.3	5.8
Primary school completed	11.8	12.1	21.3	16.8
Secondary school completed	38.2	14.4	41.7	35.3
High school or equivalent completed	26.3	39.4	7.2	30.3
College or post-graduate completed	17.0	27.4	23.5	11.8
Health care need, (%)				
Member with long-term illness, disability or frailty	4.6	4.2	3.2	3.3
Respondent with chronic illness	23.1	30.7	41.4	16.4
Hospitalization	7.2	18.0	15.4	14.5
Risk protection, (%)				
Households with no reported health insurance	40.5	41.7	75.4	42.5
Households with at least one member but not all insured	18.8	30.9	13.8	50.5
Households with all members insured	40.7	27.4	10.8	7.1
Poverty, (%)^b^				
Households classified as poor	19.1	20.5	39.0	30.6
Total annual expenditure, *median by wealth quintile* ($)^c^				
Q1 (fewest assets)	2468	2471	2673	3080
Q2	3760	4127	4103	3491
Q3	5560	5405	5097	4107
Q4	7276	6756	6838	5134
Q5 (most assets)	10,490	10,956	10,319	8220

^a^All estimates are weighted to adjust for complex survey design.

^b^Poverty line constructed using a weighted median food expenditure in each country sample (Xu, et al., 2003).

^c^Expenditures expressed in 2002/03 International Dollars.

### Estimates of economic burden from health care payments

Table 3 shows the magnitude of economic burden from out-of-pocket health care payments in the past 12 months according to the five survey measures. In each country over 50% of households reported paying for health care in the past year. The median health expenditure amount for the middle wealth quintile ranged from $37 in Malaysia to $91 in China. Malaysia had the lowest percentage of households with catastrophic spending (4.6%) and indebtedness (10.6%), while the Philippines showed the highest rates (18.6% and 30.7%, respectively). Impoverishment from health care payments, arguably the most serious form of economic burden, was between 5% and 10% in China, the Philippines, and Vietnam but lower in Malaysia (2.4%).

**Table 3:** **Household economic burden due to health care payments in past 12 months**^**a**^

	**Country**			
**Measure of economic burden**	**China**	**Malaysia**	**Philippines**	**Vietnam**
	**(n=3993)**	**(n=6095)**	**(n=10,074)**	**(n=4169)**
Any health expenditure, (%)				
Household paid for health care	53.6	56.0	58.5	56.3
Health expenditure amount, *median by wealth quintile* ($)^b^				
Q1 (fewest assets)	91	0	0	66
Q2	61	0	62	77
Q3	91	37	75	51
Q4	121	80	162	83
Q5 (most assets)	303	292	373	124
Catastrophic health expenditure, (%)^c^				
Household health expenditure/nonsubsistence expenditure ≥ 40%	16.8	4.6	18.6	13.4
Indebtedness, (%)				
Household borrowed money or sold assets to pay for health care	17.0	10.6	30.7	19.0
Impoverishment, (%)^c,d^				
Household newly classified as poor after paying for health care	5.7	2.4	9.0	7.7

^a^All estimates are weighted to adjust for complex survey design.

^b^Expenditures expressed in 2002/03 International Dollars.

^c^Variable constructed using WHO methodology (Xu, 2005).

^d^Denominator for impoverishment variable includes households not classified as poor prior to paying for health care.

### Relationships between measures of economic burden and known constructs

**Figure 1:**
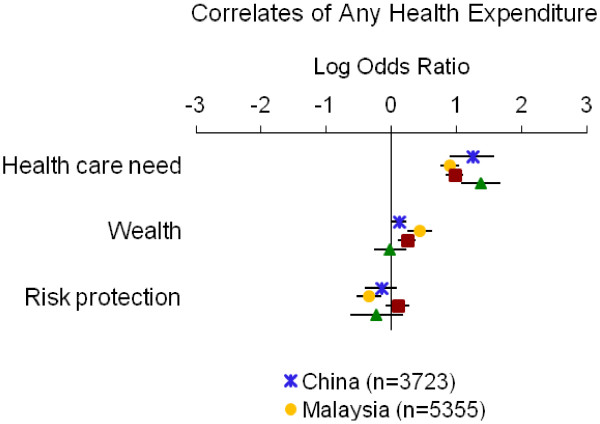
**Relationship between having any health expenditure and indicators of health care need, wealth and risk protection.** Point estimates with 95% confidence intervals shown.

**Figure 2:**
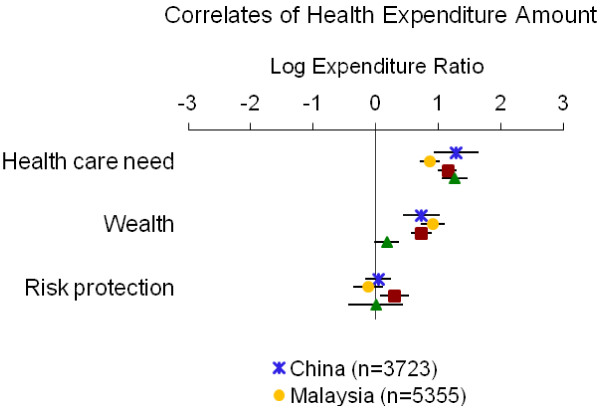
**Relationship between health expenditure amount and indicators of health care need, wealth and risk protection.** Point estimates with 95% confidence intervals shown.

**Figure 3:**
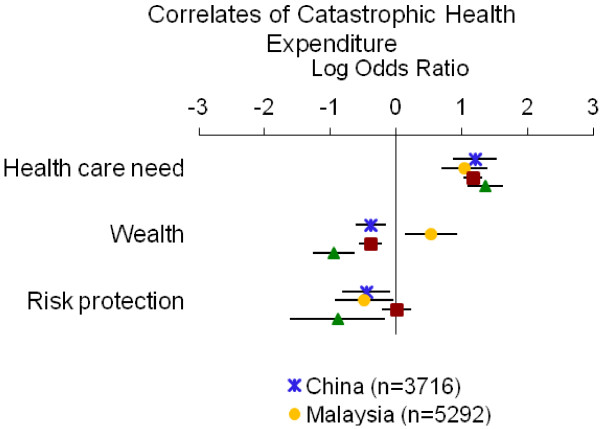
**Relationship between catastrophic health expenditure and indicators of health care need, wealth and risk protection.** Point estimates with 95% confidence intervals shown.

**Figure 4:**
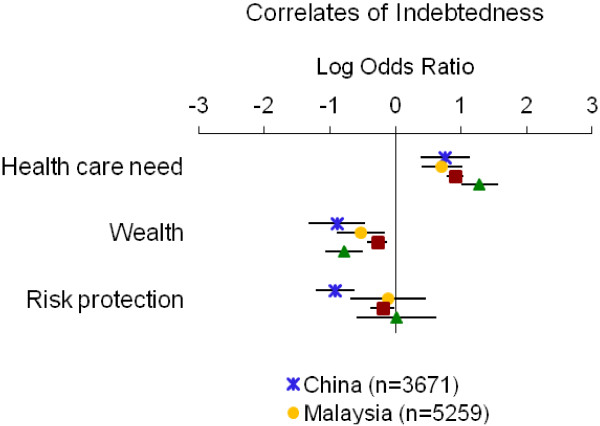
**Relationship between indebtedness and indicators of health care need, wealth and risk protection.** Point estimates with 95% confidence intervals shown.

**Figure 5:**
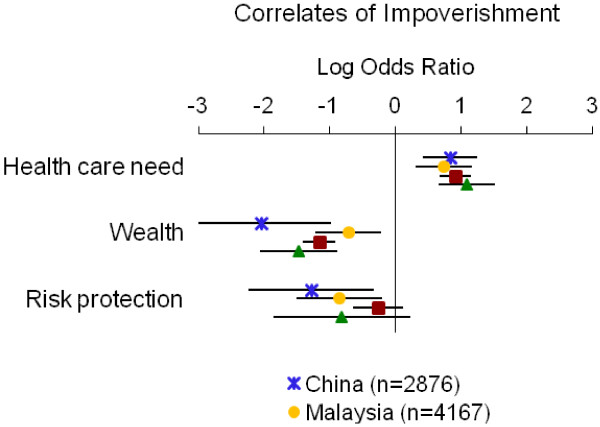
**Relationship between impoverishment and indicators of health care need, wealth and risk protection.** Point estimates with 95% confidence intervals shown.

Table 4 summarizes the directions of associations from adjusted models; detailed results for each measure of economic burden are displayed in Figures 1, 2, 3, 4 and 5. Overall, the impoverishment measure had the most consistent associations across countries and constructs that agreed with our hypotheses (12), while the indebtedness, catastrophic health expenditure, any health expenditure, and health expenditure amount measures displayed fewer consistent relationships (11, 10, 10, and 9, respectively).

**Table 4:** **Summary of associations between measures of economic burden and health care need**, **wealth and risk protection constructs**^**a**^

	**Measures of economic burden from health care payments**				
	**Direct cost**			**Direct negative consequences**	
**Construct**	**Any health expenditure**	**Health expenditure amount**	**Catastrophic health expenditure**	**Indebtedness**	**Impoverishment**
Health care need	+ / 4	+ / 4	+ / 4	+ / 4	+ / 4
Wealth	+ / 3	+ / 4	– / 3	– / 4	– / 4
Risk protection	– / 3	– / 1	– / 3	– / 3	– / 4
**Total**^**b**^	**10**	**9**	**10**	**11**	**12**

^a^Plus or minus signs reflect hypothesized positive or negative correlations. Numbers indicate tally of study countries (out of four) with associations that were consistent with hypothesized directions.

^b^Total count of associations (across countries and constructs) in which the observed matched the expected directions.

In all instances the impoverishment measure showed relationships with the indicators of health care need (i.e., hospitalization, disability or frailty, or chronic illness), wealth, and risk protection (i.e., health insurance) that were consistent with our hypotheses (Table 4, Figure 5). In each country health care need correlated positively with impoverishment (log ORs=0.74 to 1.08), while wealth and risk protection each correlated negatively with impoverishment (log ORs=−2.05 to −0.72 and −1.29 to −0.26, respectively).

The indebtedness measure exhibited the hypothesized relationships in all but one case (Table 4, Figure 4). In each country we observed positive correlations between health care need and indebtedness (log ORs=0.71 to 1.28) and negative correlations between wealth and indebtedness (log ORs=−0.89 to −0.27). Risk protection was negatively associated with indebtedness in each country (log ORs=−0.92 to −0.11) except Vietnam, where it showed a marginally positive association based on our decision rule (log OR=0.02).

The catastrophic health expenditure measure exhibited most of the hypothesized relationships with two unexpected associations of both wealth and risk protection with catastrophic spending (Table 4, Figure 3). In each country the indicators of health care need were positively associated with catastrophic expenses (log ORs=1.05 to 1.36). Wealth correlated negatively with catastrophic spending in all countries (log ORs=−0.95 to −0.38) except Malaysia (log OR=0.53). Similarly, risk protection correlated negatively with catastrophic payments in all countries (log ORs=−0.89 to −0.45) except the Philippines (log OR=0.01), where the association was marginal. Our sensitivity analyses using the 30% and 50% thresholds for determining catastrophic health expenditure showed similar results.

The measure of any health expenditure showed the hypothesized relationships in all but two cases (Table 4, Figure 1). In each country the indicators of health care need correlated positively with having any health expense (log ORs=0.90 to 1.37). Wealth showed positive associations with paying for care in every country (log ORs=0.12 to 0.44) except Vietnam, where it had a marginally negative association (log OR=−0.02). Risk protection correlated negatively with having any health expenditure in each country (log ORs=−0.34 to −0.16) except the Philippines, which showed a marginally positive association (log OR=0.10).

In several instances the health expenditure amount measure performed as expected; however, the measure’s associations with risk protection were largely inconsistent with our hypotheses (Table 4, Figure 2). In each country the indicators of health care need correlated positively with health expenditure amount (log ERs=0.87 to 1.28). Wealth also predicted greater health expenditures in all countries (log ERs=0.18 to 0.92). Contrary to our hypotheses risk protection was associated with greater health payments in the Philippines (log ER=0.30) and, marginally, in China and Vietnam (log ERs=0.04 and 0.002, respectively); risk protection predicted lower health spending only in Malaysia (log ER=−0.12).

In general we found similar results in stratified analyses (Table 5). The association between health care need and all measures of economic burden did not vary by wealth group. However, in several instances, across all outcome measures, the relationship with risk protection showed appreciable differences by wealth group; such variation was present in each country and lacked a general pattern.

**Table 5:** **Model results for each measure of economic burden**, **stratified analyses**

	**China**			**Malaysia**			**Philippines**			**Vietnam**		
	**All**	**Low**	**High**	**All**	**Low**	**High**	**All**	**Low**	**High**	**All**	**Low**	**High**

Correlates of AHE^a,b,c,f^	(n=3,723)	(n=2,269)	(n=1,454)	(n=5,355)	(n=3,194)	(n=2,161)	(n=9,558)	(n=5,723)	(n=3,835)	(n=3,218)	(n=1,907)	(n=1,311)
Wealth	1.12	---	---	1.55***	---	---	1.27***	---	---	0.98	---	---
Health care need	3.46***	3.34***	3.61**	2.46***	2.65***	2.20***	2.64***	2.62***	2.69***	3.95***	3.36***	5.11***
Risk protection	0.85	0.74	1.20	0.71**	0.61***	0.90	1.10	1.04	1.17	0.80	0.93	0.69

Correlates of HEA^a,c,d,f^	(n=3,723)	(n=2,269)	(n=1,454)	(n=5,355)	(n=3,194)	(n=2,161)	(n=9,558)	(n=5,723)	(n=3,835)	(n=3,218)	(n=1,907)	(n=1,311)
Wealth	2.08***	---	---	2.50***	---	---	2.08***	---	---	1.19	---	---
Health care need	3.61***	3.65***	2.59**	2.39***	2.67***	2.12***	3.16***	3.24***	2.94***	3.54***	3.20***	4.13***
Risk protection	1.04	1.53	0.78	0.89	0.70*	1.39*	1.35*	1.00	1.57**	1.00	0.57	1.18

Correlates of CHE^a,b,c,f^	(n=3,716)	(n=2,263)	(n=1,453)	(n=5,292)	(n=3,142)	(n=2,150)	(n=9,353)	(n=5,521)	(n=3,832)	(n=3,212)	(n=1,902)	(n=1,310)
Wealth	0.68**	---	---	1.70*	---	---	0.68***	---	---	0.39***	---	---
Health care need	3.33***	3.29***	3.46***	2.85***	3.73***	2.04*	3.20***	3.02***	3.57***	3.89***	3.27***	7.47***
Risk protection	0.64*	0.68	0.54**	0.61*	0.40**	1.00	1.01	0.95	1.07	0.41*	0.46	0.35*

Correlates of IND^a,b,c,f^	(n=3,671)	(n=2,234)	(n=1,437)	(n=5,259)	(n=3,136)	(n=2,123)	(n=9,456)	(n=5,653)	(n=3,803)	(n=2,999)	(n=1,786)	(n=1,213)
Wealth	0.41***	---	---	0.59**	---	---	0.76***	---	---	0.46***	---	---
Health care need	2.13***	2.26***	1.66	2.03***	2.27***	1.59	2.47***	2.51***	2.39***	3.61***	3.53***	3.93***
Risk protection	0.40***	0.45***	0.27**	0.90	0.99	0.61	0.82*	0.93	0.71**	1.02	1.13	0.89

Correlates of IMP^a,b,c,e,f^	(n=2,876)	(n=1,464)	(n=1,412)	(n=4,167)	(n=2,132)	(n=2,035)	(n=5,839)	(n=2,481)	(n=3,358)	(n=2,203)	(n=1046)	(n=1,157)
Wealth	0.13**	---	---	0.49**	---	---	0.31***	---	---	0.23***	---	---
Health care need	2.31***	2.08**	8.24**	2.09**	2.17**	1.85	2.51***	2.53***	2.46***	2.95***	2.45***	7.16**
Risk protection	0.28*	0.24**	1.67	0.43*	0.17**	1.14	0.77	0.80	0.74	0.44	0.63	0.11*

^a^AHE: any health expenditure; HEA: health expenditure amount; CHE: catastrophic health expenditure; IND: indebtedness; IMP: impoverishment.

^b^All estimates are weighted odds ratios and adjust for complex survey design. All models are adjusted for education, urbanicity, and household composition.

^c^ Models conducted for all households (All), households in 3 lowest wealth quintiles (Low), or households in 2 highest wealth quintiles (High).

^d^All estimates are weighted ratios of health expenditures and adjust for complex survey design (GLM specification: log link and gamma variance function). All models are adjusted for education, urbanicity, and household composition.

^e^Denominator for impoverishment analysis includes households not classified as poor prior to paying for health care.

^f^(*) P<0.05, (**) P<0.01, (***) P<0.001.

## Discussion

Many low and middle-income countries rely on out-of-pocket health care payments to help finance their national health care systems. Out-of-pocket payments can pose considerable financial hardships on households; therefore, accurate measurement of this type of economic burden is critical. Our study is the first, to our knowledge, to assess the construct validity of five common survey measures of economic burden from health care payments.

Overall, we found that all five measures correlated with at least some of the other constructs—health care need, wealth, and risk protection—in expected ways; however, the impoverishment and indebtedness measures showed the strongest evidence of construct validity. All associations with the impoverishment measure agreed with our hypotheses. The indebtedness measure showed consistent relationships in all but one case in which risk protection in Vietnam predicted a slightly greater incidence of indebtedness. Although this could reflect a limitation of the measure, we believe this finding may instead reflect unique characteristics of the Vietnam health care and financing system. For example, insured households may have been exposed to greater health expenses and resulting indebtedness than uninsured households because risk protection made them more disposed to seeking care and they then may have incurred copayments or coinsurance for covered and full payments for uncovered services, in addition to possible “under-the-table” provider fees [28]. Conversely, uninsured households may have avoided indebtedness more often by not seeking care.

The impoverishment and indebtedness measures likely performed well because they assess severe consequences of unaffordable health care payments. By definition, households with indebtedness, poor and non-poor, reported using undesirable strategies to pay for care. While these households may be protected from economic risks in the short term, their future financial stability and livelihood are threatened should they forgo basic needs to repay debts or restore essential household belongings [12]. Similarly, households facing impoverishment experienced a critical reduction in available resources after paying for health care, putting them below the poverty line. Both impoverishment and indebtedness measures therefore capture serious economic burden attributable to health care expenditures.

Our study showed that the direct cost measures demonstrated inconsistent evidence of construct validity and, absent contextual information, may have limited utility as measures of economic burden. In particular, the construction of the catastrophic health expenditure measure may lead to paradoxical results: contrary to our hypotheses, catastrophic spending in Malaysia was significantly more common among wealthier than poorer households. If Malaysia’s payment exemption scheme was successfully implemented, this finding may be a sign that many poor households were in fact protected against catastrophic spending. The relatively low prevalence of catastrophic spending in Malaysia may provide some evidence of this. However, even if the payment exemption was effective among some poor households who receive care in the public sector, the finding may not accurately reflect the experience of other Malaysian households: non-poor households that were ineligible for the exemption and which may have resources to seek higher cost private sector care may have incurred catastrophic payments in the process. We do not know to what extent meeting the catastrophic payment threshold translates into economic hardship for non-poor households. The catastrophic health expenditure measure therefore requires contextual information about policy implementation or household characteristics for interpretation.

The measures of any health expenditure and health expenditure amount also showed unexpected relationships that were difficult to interpret. For example, poorer households in Vietnam were slightly more likely to have any health expense than wealthier households. This may indicate greater exposure to user fees in public facilities, or reflect more sickness and greater need for health care among the poor. We also found that risk protection correlated positively with having any health expenditure and the health expenditure amount in several instances. This may suggest that insured households experienced, on average, greater health care payments by gaining access to care or by overusing services in response to having coverage (i.e., moral hazard), neither of which, alone, can be considered burdensome.

Our study has several potential limitations. First, our indicators for health care need, wealth, and risk protection were imperfect. Measurement error may have biased our assessments of construct validity. For example, we measured risk protection based on dichotomous self-reported insurance coverage status, although we lacked information on the specific insurance benefit design. As a result we may have misclassified as risk-protected some households whose insurance benefit conferred limited or no protection against high out-of-pocket health care payments. However, for the most part, insurance was associated with the outcome measures as hypothesized.

Second, we calculated annual expenditures for all non-hospital items using a reported 4-week expense. Because of fluctuations in household spending on health care, such annualized expenses for non-hospital items may not accurately reflect full-year expenditures. Third, the impoverishment measure excludes households that were already classified as poor before paying for health care. This measure therefore does not reflect economic burden for the most vulnerable households, whose experience may be of primary interest for research in this area. Studies that examine the impact of health care payments on the poor may alternatively focus on the indebtedness measure, which captures all households. However, impoverishment would be a crucial measure to assess health care payment effects for the near-poor whose decline into poverty may be preventable.

Finally, our assessment of construct validity used data from four unique Asia Pacific countries and may therefore not extend to other countries with incomparable health systems, living standards, or populations [13,39]. However, in the absence of a non-survey, gold standard method of validation our study sheds light on the limitations of common survey measures of economic burden and may guide other researchers in choosing an appropriate measure.

## Conclusions

For research on health care affordability, accurate measurement of economic burden from health care payments is important. We examined the construct validity of several common survey measures of economic burden and found that measures of impoverishment and indebtedness were the best indicators of this type of burden. These measures focus on direct negative consequences of high out-of-pocket payments and, in so doing, help illuminate how households experience burden. The measures of catastrophic health expenditure, any health expenditure, and health expenditure amount were less useful if contextual information is unknown. Policy-makers and researchers concerned about the economic burden of out-of-pocket health care payments should focus their attention on measures of impoverishment and indebtedness.

## Competing interests

No conflict of or competing interest declared.

## Authors’ contributions

SRR contributed to the conception and design of the study; helped to acquire, analyze, and interpret the data; drafted the manuscript; and revised the manuscript for important intellectual content. DRD contributed to the conception and design of the study; helped to acquire, analyze and interpret the data; and revised the manuscript for important intellectual content. AMZ contributed to the conception and design of the study; helped to analyze and interpret the data; and revised the manuscript for important intellectual content. SBS helped to analyze and interpret the data and revised the manuscript for important intellectual content. AKW contributed to the conception and design of the study; helped to acquire, analyze, and interpret the data; and revised the manuscript for important intellectual content. All authors read and approved the final manuscript.
